# A Case Report of a 17 cm Retropharyngeal Well-Differentiated Liposarcoma With Bilateral Neck Extension

**DOI:** 10.7759/cureus.55795

**Published:** 2024-03-08

**Authors:** Ana L Melero-Pardo, Tatiana C Pimentel-Soler, Pedro Rullán-Marín, Gustavo A Melero-Gigante

**Affiliations:** 1 School of Medicine, Universidad Central del Caribe, San Juan, PRI; 2 Otolaryngology, Veterans Affairs Hospital, San Juan, PRI

**Keywords:** otolaryngology case report, well-differentiated liposarcoma, complete tumor resection, retropharyngeal, liposarcoma

## Abstract

Liposarcomas, malignant adipocytic tumors, primarily manifest in the lower extremities and retroperitoneum, with a strikingly low incidence in the head and neck region. Symptomatology typically remains absent until the tumor attains significant size, leading to cosmetic concerns or compression-related complications. This report presents a unique case of well-differentiated retropharyngeal liposarcoma in an 81-year-old male, emphasizing diagnostic challenges, management strategies, and the crucial role of Mouse double minute 2 (MDM2) fluorescence in situ hybridization (FISH) analysis in confirmation.

The patient exhibited dysphagia attributed to a retropharyngeal mass, prompting suspicion of malignancy. Diagnostic assessments, including flexible laryngoscopy and percutaneous tru-cut biopsy, highlighted unique features such as atypical nuclear features in adipocytes. MDM2 FISH analysis definitively confirmed the diagnosis by detecting MDM2 gene amplification.

The rarity of retropharyngeal liposarcomas complicates diagnosis, often leading to confusion with benign lesions. Surgical excision, the mainstay of treatment, varies based on tumor size and extension. In this case, a left neck dissection via a hockey stick incision successfully resected a 17 cm well-differentiated liposarcoma. Pathologic analysis revealed focal involvement of resection margins, with no complications or vocal cord damage.

In conclusion, retropharyngeal liposarcomas pose diagnostic challenges, warranting reliance on MDM2 FISH analysis for accurate confirmation. Early surgical intervention, guided by tumor size and extension, is paramount for optimal outcomes in managing these rare tumors. This case underscores the significance of a detailed surgical approach in achieving successful outcomes for retropharyngeal liposarcomas.

## Introduction

Liposarcomas are malignant adipocytic tumors that commonly occur in the lower extremities and retroperitoneum. The incidence of liposarcoma in the head and neck region is extremely low, approximately 1.8-6.2% [1]. Patients with liposarcoma typically do not display symptoms unless the tumor reaches a significant size, which can result in cosmetic disfigurement or compression-related complications. The specific symptoms vary based on the tumor’s anatomic location and its size. Due to the paucity of data regarding liposarcomas in the head and neck region, the treatment approach for these tumors has primarily relied on the collective experience gained from managing liposarcomas in the limbs and retroperitoneal areas. The main treatment for liposarcoma is complete surgical excision with adequate margins [2]. This report details a case of retropharyngeal well-differentiated liposarcoma, emphasizing diagnostic challenges, management strategies, and the significance of Mouse double minute 2 (MDM2) fluorescence in situ hybridization (FISH) analysis for diagnosis confirmation. 

This article was previously presented as a meeting abstract at the 2023 Abstract Competition for the American College of Physicians Puerto Rico Chapter on November 18, 2023, the 22nd Annual Convention of the College of Medical-Surgeons of Puerto Rico on December 2, 2023, and the 73rd Annual Meeting of the American College of Surgeons Puerto Rico Chapter on February 24, 2024.

## Case presentation

**Figure 1:**
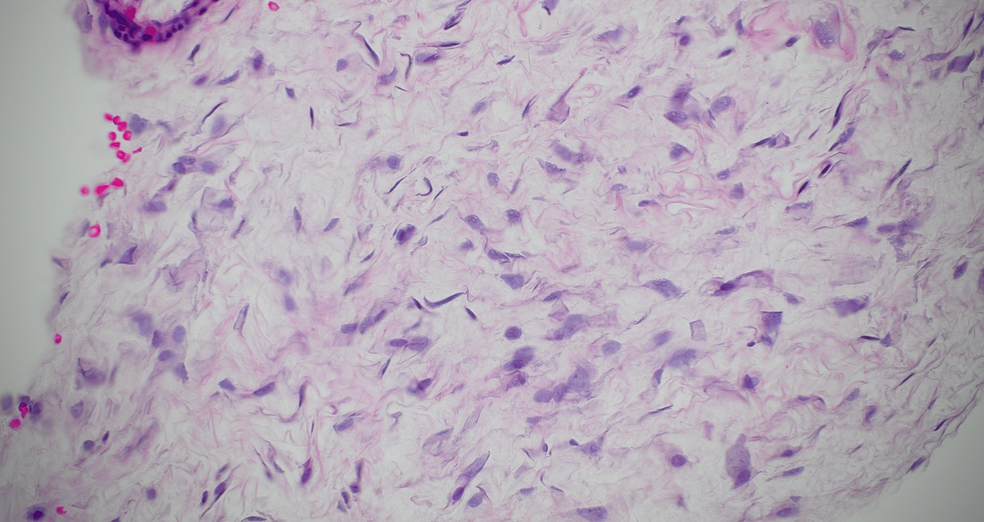
Hematoxylin and eosin (H&E) stain pathology image (40x magnification) showcases a percutaneous tru-cut biopsy revealing well-differentiated adipocytes with distinctive atypical nuclear features, providing critical histopathological insights into the unique characteristics of the well-differentiated retropharyngeal liposarcoma.

**Figure 2:**
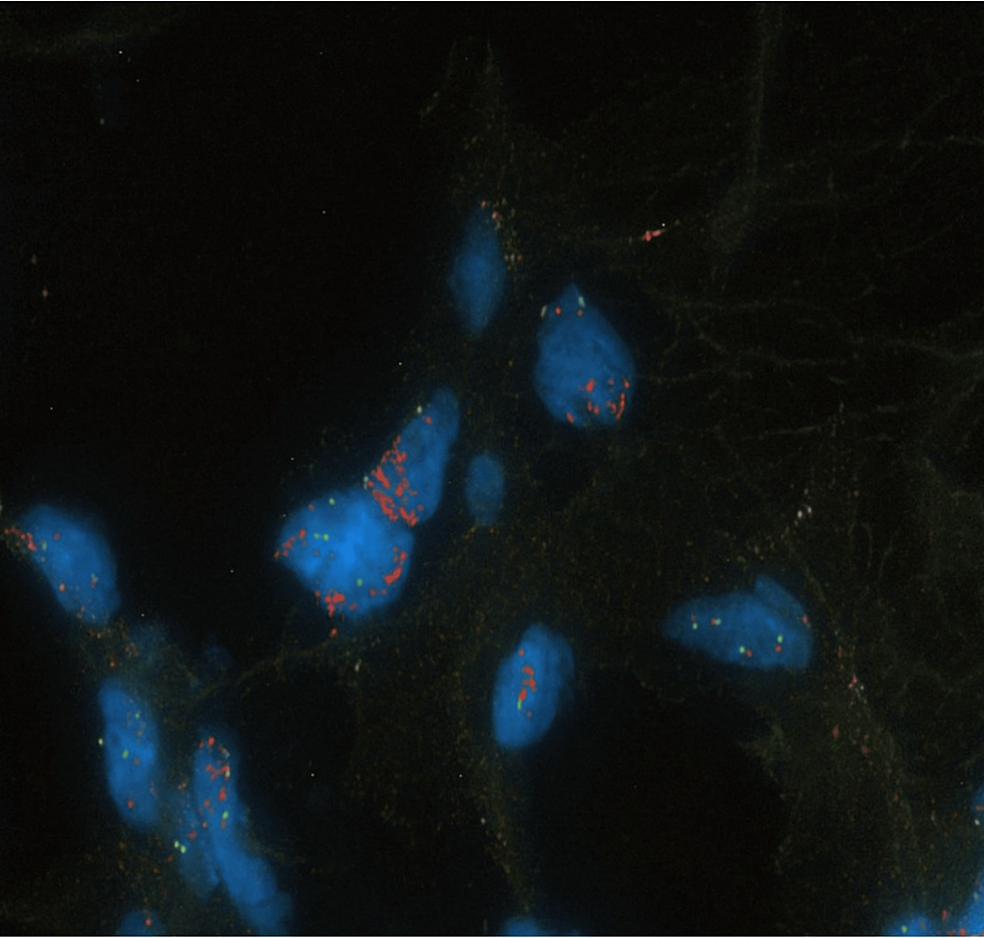
The Mouse double minute 2 (MDM2) fluorescence in situ hybridization (FISH) analysis image confirms the diagnosis of liposarcoma through the identification of MDM2 gene amplification, a hallmark molecular alteration in these tumors.

**Figure 3:**
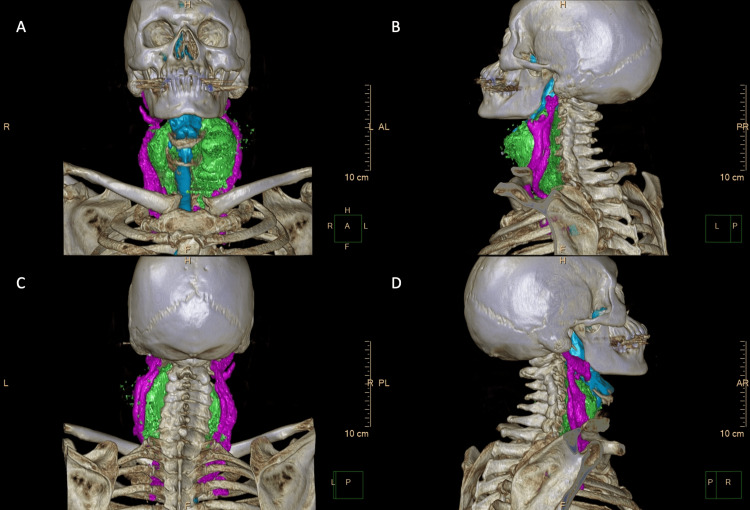
Three-dimensional CT reconstruction of liposarcoma. Anterior view (A), left lateral view (B), posterior view (C), and right lateral view (D) showing tumor (in green), airway structures (in blue), and vasculature (in pink).

An 81-year-old male Vietnam veteran presented with complaints of shortness of breath and dysphagia due to a large retropharyngeal mass. The mass was identified on head computed tomography (CT) imaging, findings that were not previously identified on CT imaging performed 33 months prior, and thus prompting concern for malignancy. Physical examination revealed a 7 x 6 cm soft tissue mass in the left neck with tracheal deviation to the right, slight fullness in the right paratracheal area, and hypopharyngeal compression. Flexible laryngoscopy demonstrated medial displacement of the left lateral pharynx, extrinsic compression, obstruction of the lateral pharyngo-epiglottic fold, pyriform sinus, and a check valve effect against the epiglottis. A percutaneous tru-cut biopsy revealed well-differentiated adipocytes with atypical nuclear features, however lipoblasts were not identified (Figure 1). MDM2 FISH analysis confirmed MDM2 gene amplification, confirming the diagnosis of liposarcoma (Figure 2). Radiology created a three-dimensional CT reconstruction of the tumor which allowed for a clearer depiction of disease morphology, enhancing the present compression and displacement of the hypopharyngeal wall, bilateral displacement of the carotid arteries and jugular veins (Figure 3A-3D). 

## Discussion

Retropharyngeal liposarcomas are extremely rare and often challenging to diagnose due to their uncommon location and variable radiographic appearances. These tumors can be mistaken for benign lipomas or other soft tissue neoplasms. In our case, the large mass identified on CT imaging and the smooth anterior bowing and displacement of the retropharyngeal wall raised suspicion for malignancy. The final diagnosis was established through MDM2 FISH analysis, a critical tool for distinguishing liposarcomas from benign lipomatous lesions.

Surgical intervention is the primary treatment for retropharyngeal liposarcomas and surgical approach depends on tumor size and extension into neighboring structures. Literature reports cases of retropharyngeal liposarcomas that have been resected via an open anterior neck dissection, cervical or transoral approach [3-9]. In our case, the surgical approach involved a series of meticulous procedures aimed at ensuring optimal management and preservation of vital structures. Initially, due to the complexity of the airway management, a tracheostomy was performed under local anesthesia, sedation, and continuous cardiac monitoring to secure the airway. Subsequently, once the airway was controlled the patient was induced under general anesthesia and intubated endotracheally using a neuromonitoring endotracheal tube for recurrent laryngeal nerve monitoring during surgery. Following this, the tracheostomy was removed, and the surgery proceeded under endotracheal intubation. The tumor resection commenced with a left neck dissection through a hockey stick incision. A subplatysmal flap was meticulously elevated to expose the sternocleidomastoid muscle and submandibular gland. The mass was then meticulously dissected away from surrounding structures, including the sternocleidomastoid, digastric, and strap muscles, with particular attention paid to identifying and safeguarding the integrity of the spinal accessory nerve, left superior laryngeal nerve, left recurrent laryngeal nerve, and left hypoglossal nerves. To achieve inferior release, the omohyoid muscle was carefully reflected inferiorly. Subsequently, the right aspect of the mass was approached via the retropharyngeal space, where meticulous blunt and sharp dissections were employed to completely excise the retropharyngeal mass, ensuring thorough removal while minimizing damage to adjacent structures.

Surgical pathology confirmed a well-differentiated liposarcoma measuring 17 cm in the greatest dimension and focally involving the resection margins. Examination of regional lymph nodes revealed an absence of tumor invasion, which contrasts with documented instances in the literature where liposarcomas have been associated with secondary involvement of lymph nodes [10]. No perioperative complications were reported. Following the surgical intervention, the patient underwent a comprehensive course of post-operative radiotherapy involving external beam radiation treatment employing 6 MV photons at a dosage of 180 cGy per fraction administered over 37 sessions with a total dose of 6660 cGy. Subsequent evaluation via post-radiotherapy CT scan of the neck revealed an absence of detectable disease. Additionally, during the one-year postoperative follow-up visit to the otolaryngology clinic, there was no indication of disease recurrence.

## Conclusions

Retropharyngeal liposarcomas are rare tumors and MDM2 FISH analysis is a valuable diagnostic tool, allowing for accurate differentiation from benign lipomatous lesions. Early diagnosis and surgical resection are crucial for achieving optimal outcomes in these cases. This case highlights the surgical approach to effectively manage retropharyngeal liposarcomas and achieve favorable patient outcomes.
